# Functional Characterization of Six Eukaryotic Translation Initiation Factors of *Toxoplasma gondii* Using the CRISPR-Cas9 System

**DOI:** 10.3390/ijms25147834

**Published:** 2024-07-17

**Authors:** Yong-Jie Kou, Jin Gao, Rui Li, Zhi-Ya Ma, Hany M. Elsheikha, Xiao-Jing Wu, Xiao-Nan Zheng, Meng Wang, Xing-Quan Zhu

**Affiliations:** 1 Laboratory of Parasitic Diseases, College of Veterinary Medicine, Shanxi Agricultural University, Taigu, Jinzhong 030801, China; kouyongjie1997@163.com (Y.-J.K.); jingao2022@163.com (J.G.); mzy16635048046@126.com (Z.-Y.M.); wuxiaojing2017@163.com (X.-J.W.); zhengxiaonan8889@126.com (X.-N.Z.); 2 State Key Laboratory for Animal Disease Control and Prevention, Key Laboratory of Veterinary Parasitology of Gansu Province, Lanzhou Veterinary Research Institute, Chinese Academy of Agricultural Sciences, Lanzhou 730046, China; 17393145594@163.com; 3 Faculty of Medicine and Health Sciences, School of Veterinary Medicine and Science, Sutton Bonington Campus, University of Nottingham, Loughborough LE12 5RD, UK; hany.elsheikha@nottingham.ac.uk; 4 Institute of Urban Agriculture, Chinese Academy of Agricultural Sciences, Chengdu 610213, China

**Keywords:** *Toxoplasma gondii*, eukaryotic protein translation initiation factors, CRISPR-Cas9, differentiation

## Abstract

Eukaryotic translation initiation factors (eIFs) are crucial for initiating protein translation and ensuring the correct assembly of mRNA-ribosomal subunit complexes. In this study, we investigated the effects of deleting six eIFs in the apicomplexan parasite *Toxoplasma gondii* using the CRISPR-Cas9 system. We determined the subcellular localization of these eIFs using C-terminal endogenous tagging and immunofluorescence analysis. Four eIFs (RH::315150-6HA, RH::286090-6HA, RH::249370-6HA, and RH::211410-6HA) were localized in the cytoplasm, while RH::224235-6HA was localized in the apicoplast. Additionally, RH::272640-6HA was found in both the basal complex and the cytoplasm of *T. gondii*. Functional characterization of the six RHΔ*eIFs* strains was conducted using plaque assay, cell invasion assay, intracellular growth assay and egress assay in vitro, and virulence assay in mice. Disruption of five eIF genes (RHΔ*315150*, RHΔ*272640*, RHΔ*249370*, RHΔ*211410*, and RHΔ*224235*) did not affect the ability of the *T. gondii* RH strain to invade, replicate, form plaques and egress in vitro, or virulence in Kunming mice (*p* > 0.05). However, the RHΔ*286090* strain showed slightly reduced invasion efficiency and virulence (*p* < 0.01) compared to the other five RHΔ*eIFs* strains and the wild-type strain. The disruption of the TGGT1_286090 gene significantly impaired the ability of tachyzoites to differentiate into bradyzoites in both type I RH and type II Pru strains. These findings reveal that the eukaryotic translation initiation factor TGGT1_286090 is crucial for *T. gondii* bradyzoite differentiation and may serve as a potential target for drug development and an attenuated vaccine against *T. gondii*.

## 1. Introduction

*Toxoplasma gondii* is a widely distributed protozoan parasite capable of infecting a broad range of animals and humans [1,2]. Upon infection, rapidly dividing tachyzoites disseminate throughout various tissues and organs in the host [3]. Some tachyzoites evade the host’s immune response, differentiating into latent bradyzoites enclosed within dormant tissue cysts [1]. In immunocompetent individuals, *T. gondii* infections are typically asymptomatic. However, the parasite can cause severe infections in individuals with weakened immunity, such as those living with AIDS or organ transplant recipients [4]. In these cases, bradyzoites can convert to actively proliferating tachyzoites, leading to serious conditions such as encephalitis, retinitis, myocarditis, and pneumonia, potentially resulting in death [5]. Additionally, *T. gondii* infection in pregnant women can lead to congenital toxoplasmosis causing severe health complications in the fetus [3,6].

Tachyzoites can be induced to differentiate into bradyzoites under stress conditions, such as an alkaline environment [7]. This transformation process involves converting the parasitophorous vacuole membrane (PVM) into a highly glycosylated cyst wall, accumulating starch granules within bradyzoites, and marked changes in gene and protein expression [8,9,10]. These findings suggest that changes in mRNA translation are crucial for producing proteins necessary for the parasite’s genome reprogramming to form bradyzoites.

Using Cas9-mediated screening and single-cell analysis, researchers identified a master gene BFD1 that regulates *T. gondii* differentiation [11]. The ApiAP2 family of transcription factors in the Apicomplexa protozoa regulates gene expression by binding to specific genes. AP2IX-4 and AP2IX-9 act as negative regulators, controlling the expression of bradyzoite genes during tachyzoite differentiation [12,13]. These studies highlight the importance of transcriptional regulation in *T. gondii* development and differentiation. Although translational control is crucial for phenotypic differentiation and other developmental processes in many parasitic protozoa [14,15], the regulatory mechanisms underlying *T. gondii* differentiation at the translation level remain incompletely understood. 

Eukaryotic translation initiation factors (eIFs) ensure the correct formation of the mRNA-ribosome subunit complex and initiation of protein translation in eukaryotic cells [16]. Previous studies have identified eIFs involved in the translation initiation in parasites, demonstrating their role in regulating genes responsible for nutrient uptake, stress response, growth, and differentiation [17,18]. The eukaryotic initiation factor 2α (eIF2α) is involved in the translation initiation in parasites such as *Plasmodium* and *T. gondii* [17,19]. In response to stress, eIF2α phosphorylation increases, reducing overall protein synthesis while prioritizing specific mRNA translation to help the parasite to adapt to different environments [17]. Despite their significance, the functions of *T. gondii* eIFs remain largely unexplored.

In this study, we investigated the roles of six eIFs (TGGT1_315150, TGGT1_272640, TGGT1_249370, TGGT1_211410, TGGT1_224235, and TGGT1_286090), selected based on their CRISPR-based phenotypic values, in the lytic cycle and virulence of *T. gondii* using the CRISPR-Cas9 system. We employed C-terminal endogenous tagging to determine the subcellular localization of these six eIFs in both the tachyzoite and bradyzoite stages. We assessed the pathogenicity of six RHΔ*eIFs* mutant strains by employing assays examining plaque formation, host cell invasion, intracellular growth, calcium ionophore-induced egress in vitro, and virulence in mice. Our findings indicate that five eIF genes are dispensable for *T. gondii* tachyzoite growth, while TGGT1_286090 significantly impacts tachyzoite invasion in vitro and virulence in vivo. Furthermore, TGGT1_286090 plays a crucial role in bradyzoite differentiation in both type I RH and type II Pru strains in vitro.

## 2. Results

### 2.1. Subcellular Localization of the Six eIFs in Tachyzoites and Bradyzoites of T. gondii

**Figure 1:**
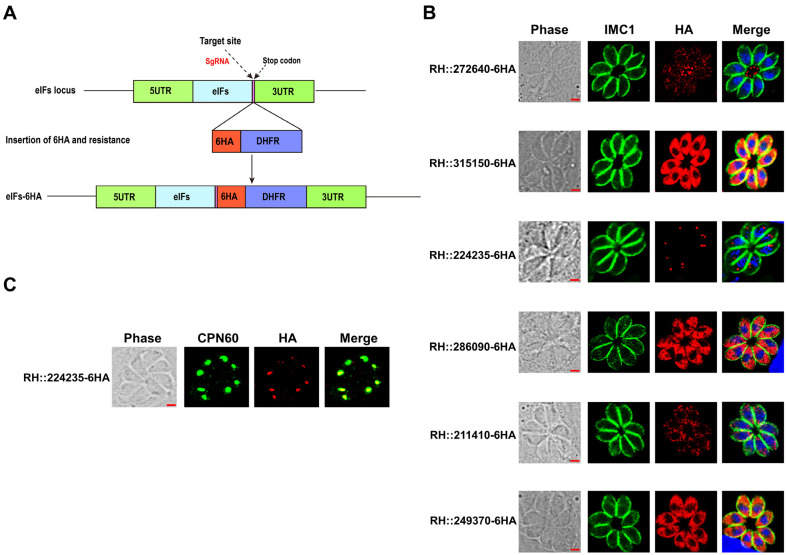
Subcellular localization of six eIF genes in the tachyzoites of *Toxoplasma gondii* (**A**) Schematic diagram illustrating C-terminal tagging of eIFs using the CRISPR-Cas9 technique. (**B**) RH::eIFs-HA epitope-tagged strains infected HFF cells for 24 h, stained with anti-IMC1 (green) and anti-HA (red) antibodies. Cell nuclei were counter-stained with DAPI labeling (blue). Scale bars, 2 μm. (**C**) Co-localization of RH::224235-HA with the apicoplast marker protein CPN60 (green).

We selected six eIF genes with higher CRISPR-based phenotypic values to examine their localization in *T. gondii* type I RH strain. Each eIF gene was tagged with a 6 × HA label at its endogenous locus using the CRISPR-Cas9 technique (Figure 1A). The integration of the 6 × HA-tagged epitope was confirmed by PCR and DNA sequencing (Figure S1A). Analysis of the six eIFs isolated from tachyzoites confirmed the integration of endogenous epitope tags and the presence of these six eIFs in the RH strain (Figure S1B). Western blot analysis verified the presence and size of the eIFs, band had the same size predicted in ToxoDB (http://toxodb.org accessed on 12 July 2024), and only TGGT1_249370 and TGGT1_224235 were different from the predicted sizes. To determine the subcellular localization of the eIFs in tachyzoites of *T. gondii*, HFF cells were infected with RH::eIFs-6HA for 24 h, and stained with mouse anti-HA and rabbit anti-IMC1 antibodies. Immunofluorescence assay (IFA) results showed that five eIFs (RH::315150-6HA, RH::286090-6HA, RH::211410-6HA, RH::272640-6HA, and RH::249370-6HA) genes were localized in the cytoplasm, while RH::272640-6HA also was observed in the basal complex [20] (Figure 1B). The RH::224235-6HA was localized in the apicoplast, and colocalized with the apicoplast marker protein CPN60 (Figure 1C). To determine the location of the six eIFs in the in vitro-induced cyst, fresh tachyzoites were incubated in an alkaline medium (pH 8.2) for 72 h. Bradyzoites-containing cysts were detected by *Dolichos biflorus agglutinin* (DBA) staining, which stains N-acetyl galactosamine on the bradyzoite cyst wall. After 72 h post-differentiation, the localization of the six eIFs in the cysts was consistent with their localization in tachyzoites (Figure S1C).

### 2.2. Construction of Six eIFs Gene Knockout Strains by CRISPR-Cas9

**Figure 2:**
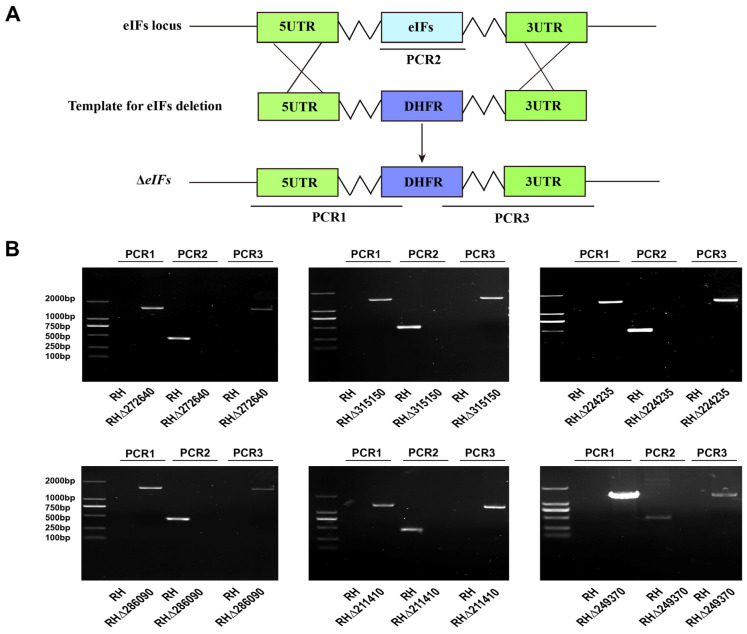
Construction of eIFs knockout strains. (**A**) CRISPR-Cas9-mediated homologous recombination was used to construct six eIF knockout strains. (**B**) Diagnostic PCRs confirmed the deletion of eIF genes in *T. gondii* RH type I strain. PCR1 and PCR3 were performed to assess the integration of a homologous fragment at the 5′ and 3′ ends of eIF genes, respectively, whereas PCR2 was used to confirm the disruption of eIF genes.

To investigate the biological roles of the six eIFs in the *T. gondii* type I RH strain, we used CRISPR-Cas9 technology to disrupt their coding regions. A homology arm replaced each eIF gene coding region with the 5HR-DHFR-3HR through recombination (Figure 2A). Single positive strains were screened via limiting dilution and pyrimethamine selection. As shown in Figure 2, the coding sequence of the eIFs gene was replaced by DHFR sequence, so the fragment could not be amplified in PCR2. In contrast, 500–700 bp fragments can be amplified from WT strains in PCR2. DHFR fragments were successfully inserted into the eIF gene locus of deletion strains, and therefore 1300–1500 bp fragments could be amplified in PCR1 and PCR3. There was no amplification product in PCR1 and PCR3 due to the absence of DHFR fragment in the WT strains. Diagnostic PCR confirmed the complete disruption of the eIF genes, revealing a ~500–700 bp fragment in WT strains, but not in knockout (KO) strains. Additionally, diagnostic PCR1 and PCR3 confirmed successful gene replacement by amplifying ~1500 bp fragments in each KO strain, not present in WT strains (Figure 2B). RT-PCR verified the successful construction of eIFs KO strains (Figure S2A).

### 2.3. Deletion of TGGT1_286090 Gene Affects the Growth of T. gondii Type I RH Strain

**Figure 3:**
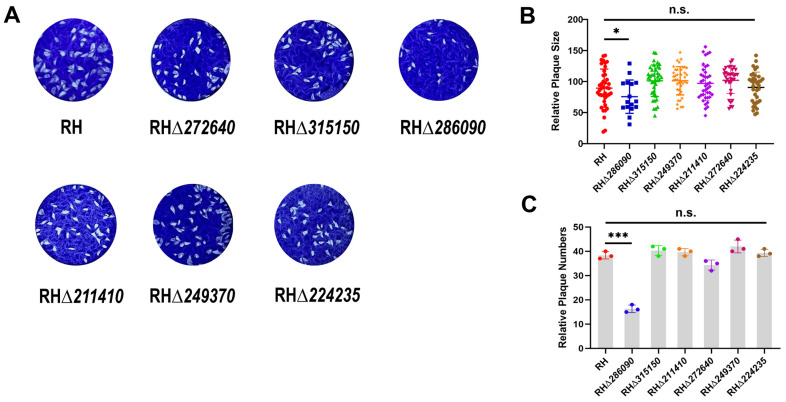
Effects of six RHΔ*eIF* strains on the growth of *T. gondii*. (**A**) The knockout of the other five eIFs genes did not influence tachyzoites growth. (**B**) Plaque assay analysis of RHΔ*286090* strains and WT strain showed that the absence of TGGT1_286090 gene inhibited the tachyzoite growth. (**C**) Comparatively, the RHΔ*286090* strain showed marked reduction in the quantity of the plaques, in contrast to WT strain (*p* < 0.01). On the other hand, the other five RHΔ*eIF* strains did not exhibit any notable differences when compared to the WT strain (*p* > 0.05). Results are shown as the means and standard deviations from three independent experiments. *, *p* < 0.05, ***, *p* < 0.001, n.s., not significant.

To assess the impact of deleting six eIF genes on the survival of RH strains, plaque assays were performed on HFF cell monolayers in 12-well plates and the plaque number and size were analyzed (see Figure 3A). Results showed no significant differences in plaque size or number between five RHΔ*eIF* mutants (RHΔ*315150*, RHΔ*272640*, RHΔ*249370*, RHΔ*211410*, RHΔ*224235*) and the WT strain (*p* > 0.05) (refer to Figure 3B,C). However, the RHΔ*286090* strain exhibited a significant reduction in plaque size and number (*p* < 0.01), indicating that TGGT1_286090 deletion partially affects the *T. gondii* lytic cycle.

**Figure 4:**
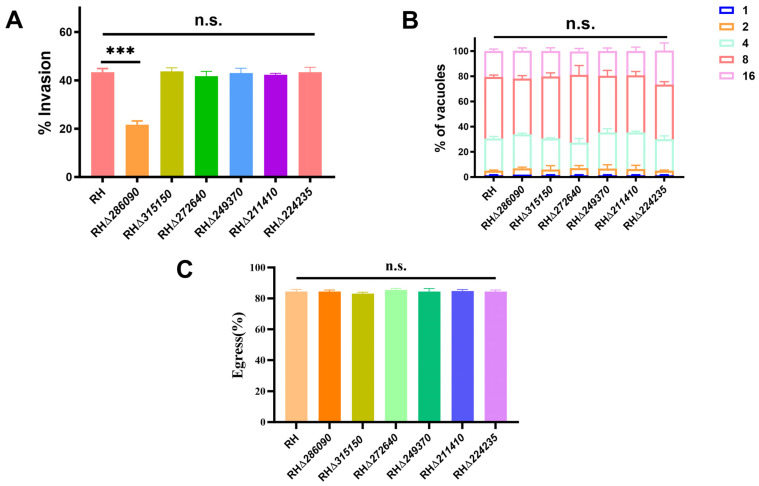
Intracellular replication, invasion, and egress assay of eIF knockout and WT strains in vitro. (**A**) The invasion efficiency of RHΔ*286090* strain exhibited a notable decrease compared to WT strain (*p* < 0.001). (**B**) At 24 h post-infection by RHΔ*eIF* and the WT strains, the number of PVs containing 1, 2, 4, 8, and 16 tachyzoites was quantified. Data represent the averages ± standard deviations of 3 independent experiments with at least 100 PVs/experiment. The intracellular replication did not show significant differences between the knockout strains of the six eIF genes and WT strain (*p* > 0.05). (**C**) The egress efficiency between the six RHΔ*eIF* strains and WT strain showed no significant differences (*p* > 0.05). ***, *p* < 0.001, n.s., not significant.

Subsequent analyses of invasion, replication, and egress of the six RHΔ*eIF* strains showed no significant differences in invasion efficiency (*p* > 0.05) for five strains compared to WT, except RHΔ*286090*, which had decreased invasion efficiency (*p* < 0.01) (Figure 4A). Results also showed no significant differences in the intracellular replication (*p* > 0.05) between the six RHΔ*eIF* strains and WT strain (Figure 4B). Egress efficiency was also similar across the six RHΔ*eIF* strains and WT strain (*p* > 0.05) (Figure 4C). These results suggested that TGGT1_286090 affected *T. gondii* growth.

**Figure 5:**
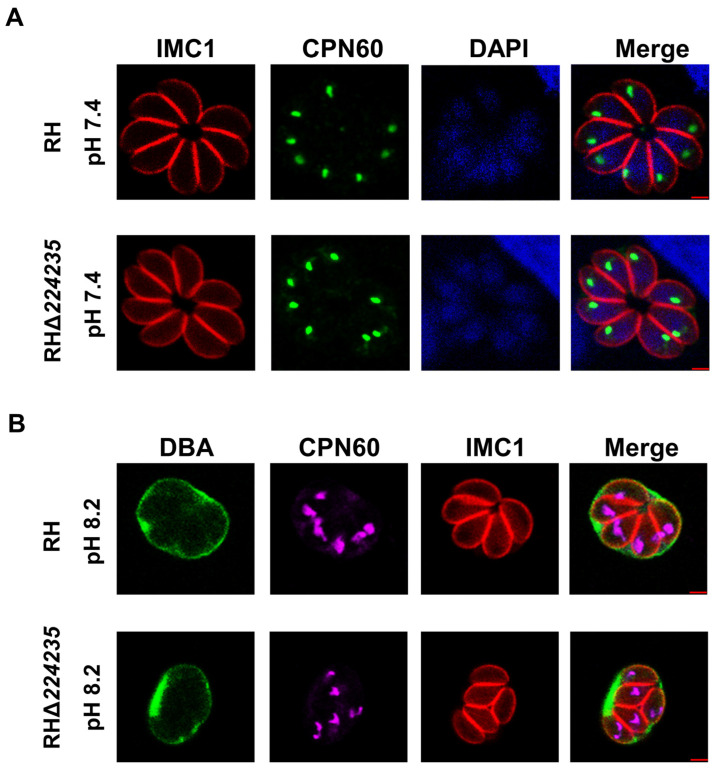
Effect of RHΔ*224235* on apicoplast morphology. (**A**) TGGT1_224235 deletion did not affect apicoplast morphology in (**A**) tachyzoites or (**B**) bradyzoites.

Because the TGGT1_224235 gene was localized in the apicoplast, we sought to evaluate the impact of the RHΔ*224235* strain on the apicoplast morphology. The IFA results showed that TGGT1_224235 deletion did not affect apicoplast morphology in the tachyzoites (Figure 5A) and bradyzoites (Figure 5B).

### 2.4. Deletion of TGGT1_286090 Gene Slightly Attenuated the Virulence of Type I RH Strain in Mice

**Figure 6:**
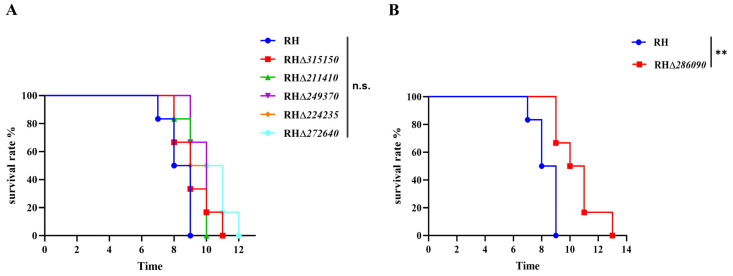
Virulence of the six RHΔ*eIF* and WT strains. (**A**) Six mice per group were injected intraperitoneally with 100 tachyzoites of each strain. Mouse survival was monitored until reaching their humane endpoints (n.s., not significant). (**B**) Six mice per group were intraperitoneally injected with 100 tachyzoites of RHΔ*286090*, and the survival curves indicated that deletion of TGGT1_286090 gene attenuated the virulence of *T. gondii* (*p* < 0.01). **, *p* < 0.01.

To assess the role of eIFs in *T. gondii* virulence, 100 tachyzoites of RHΔ*eIFs* or WT strains were intraperitoneally injected into Kunming mice. Results showed no differences in survival between mice infected with each of the five RHΔ*eIF* strains and WT strain, with all reaching their humane endpoints within 9–12 days (Figure 6A). However, mice infected with RHΔ*286090* strain showed clinical signs at 7 days and reached humane endpoints within 13 days (Figure 6B), indicating that TGGT1_286090 deletion slightly attenuated RH strain virulence (*p* < 0.01).

### 2.5. Deletion of TGGT1_286090 Gene Impeded Bradyzoite Differentiation In Vitro

**Figure 7:**
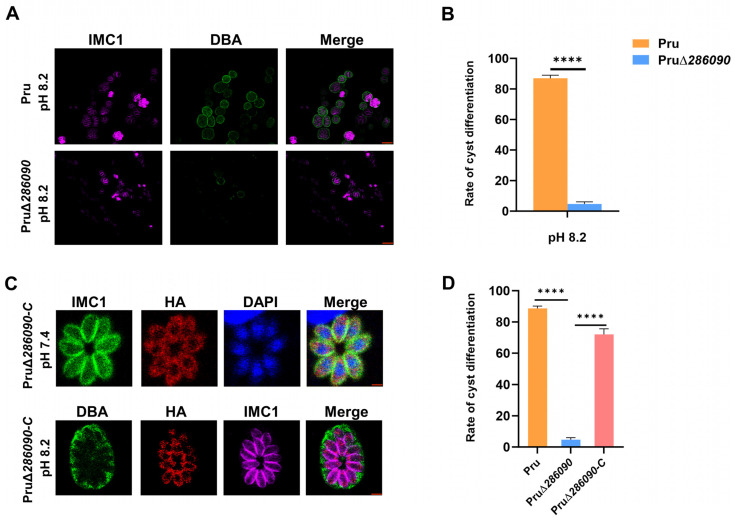
Role of TGGT1_286090 gene in cyst formation. (**A**) In vitro cysts were formed by Pru and PruΔ*286090* strains. The HFFs were infected by both strains, and bradyzoite differentiation was initiated by exposure to ambient air and pH 8.2 for 3 days. The cells were stained with IMC1 antibodies (pink) and DBA-FITC (green). Cell nuclei were counter-stained with DAPI labeling (blue). Scale bars, 5 μm. (**B**) Examination of cyst conversion rates in the Pru and PruΔ*286090* strains revealed that based on counting at least 100 vacuoles per experiment, they were categorized as either cyst wall-positive vacuoles (DBA-positive) or normal vacuoles (DBA-negative). The means and standard deviations were calculated from 3 independent experiments. The conversion ratio between the Pru and PruΔ*286090* strains were significantly different (*p* < 0.001; Student’s *t*-test). (**C**) By complementing PruΔ*286090*, TGGT1_286090 gene was consistent with the previous location, indicating PruΔ*286090-C* strain was successfully constructed. (**D**) Compared with PruΔ*286090*, the number of DBA-positive staining in PruΔ*286090-C* strain was significantly increased, indicating the ability of the complemented strain to restore cyst formation. ****, *p* < 0.0001.

The conversion between the tachyzoite and bradyzoite stages is a crucial adaptation strategy in *T. gondii* [21]. To investigate the role of six eIFs in bradyzoite differentiation induced by alkaline stress, HFFs monolayers infected with six RHΔ*eIF* strains were examined by DBA staining after 72 h of culture in alkaline conditions. By comparing the rate of cyst formation with the WT strain, the results showed that bradyzoite differentiation of the five RHΔ*eIFs* strains was comparable to the WT strain, except for RHΔ*286090*, which showed a significant decline in DBA-positive staining (Figure S2B). We also constructed a PruΔ*286090* strain using CRISPR-Cas9-mediated homologous recombination. After 72 h in alkaline conditions, HFF monolayers infected by PruΔ*286090* strains were stained with DBA (Figure 7A). PruΔ*286090* strains showed impaired cyst formation, evidenced by lack of positive DBA staining (*p* < 0.001) (Figure 7B). To determine whether the loss of TGGT1_286090 impacts parasite differentiation, we constructed a complemented strain PruΔ*286090*::HA-286090 (PruΔ*286090*-C) in PruΔ*286090* strain. We observed a consistent localization of the TGGT1_286090 gene (Figure 7C). Complementation of the TGGT1_286090 gene in PruΔ*286090* (PruΔ*286090*-C) restored cyst formation, showing a significant increase in DBA-positive staining compared to PruΔ*286090* (*p* < 0.001) (Figure 7D).

## 3. Discussion

The transition between tachyzoites and bradyzoites is a crucial event in *T. gondii* pathobiology, enabling the parasite to adapt to external stressors [9,22]. In this study, we selected six eIF genes with high CRISPR phenotype values, and successfully constructed six eIF gene knockout strains to characterize their localization and biological roles in *T. gondii*. Our results demonstrated that RH::315150-6HA, RH::286090-6HA, RH::249370-6HA, and RH::211410-6HA were localized in the cytoplasm, while RH::224235-6HA was found in the apicoplast. Additionally, RH::272640-6HA was detected in both the cytoplasm and the basal complex. Western blotting revealed specific bands corresponding to predicted sizes, with varying brightness indicating inconsistent abundance of eIF gene expression during the tachyzoite lytic cycle. The actual sizes of TGGT1_249370 and TGGT1_224235 differed from predictions, suggesting potential protein modifications.

The eIFs are involved in forming a complex for translation initiation and regulating protein production speed in response to different cellular cues [23]. The phosphorylation of the eIF2α subunit at the Ser51 site, in response to stress, is a well-recognized process controlling the start of translation. eIF2α kinases integrate various stress signals into a unified pathway, and similar eIF2α phosphorylation mechanisms exist in *T. gondii*. Under different external stress conditions, distinct eIF2α kinases promote eIF2α phosphorylation, aiding the parasite’s adaptation to stressors, such as ER stress, oxidative stress, and amino-acid starvation [24,25,26]. These studies indicate that decreased translation levels conserve energy and nutrients, giving cells more time to adjust to stress.

Among the six selected eIF genes, we did not find phosphorylation modifications similar to eIF2α, suggesting that it may have different biological functions, modification mechanisms, or involvement in other stages of protein translation. By constructing deletion strains, we evaluated the effects of the six eIF genes on *T. gondii* replication, intracellular growth, egress, invasion and virulence. Among the six eIF-deficient strains, the deletion of the TGGT1_286090 gene partially attenuated *T. gondii* growth and virulence. However, the specific mechanisms by which TGGT1_286090 as a translation initiation factor affects protein translation remain unknown. Despite the partial impact on *T. gondii*, TGGT1_286090 has potential as a base strain for further double-gene knockouts, providing avirulent strains for developing attenuated live vaccines against *T. gondii*.

eIF4E, a cap-binding protein, precisely recognizes the 5′-terminal start sites of mRNA [27]. In the present study, we generated eIF4E-deficient strains (RHΔ*315150*) and found that eIF4E deletion did not affect *T. gondii* growth, replication, invasion, or virulence. Recently, three eIF4E orthologs (4E1, 4E2, and 4E3) were identified in *T. gondii*, with only eIF4E1 binding to m^7^G-mRNA of the 5′-terminus [28]. Consistent with our findings, knockdown of TGGT1_315150 (eIF4E2) had no effect on *T. gondii*, likely due to its inability to recognize m^7^G-mRNA of 5′-terminus. However, eIF4E1 deficiency inhibited tachyzoite growth and triggered spontaneous bradyzoite cyst formation without external stress, indicating its crucial role in the translational regulation and its importance for the latent and persistent form of the parasite.

We examined the differentiation ability of six RHΔ*eIF* strains into bradyzoites. Our results showed that five RHΔ*eIF* strains did not differ in their capacity to convert into bradyzoites in vitro. However, RHΔ*286090* exhibited a significant defect in bradyzoite differentiation, suggesting a potentially critical role for TGGT1_286090 in *T. gondii* differentiation. Due to the high virulence and weak differentiation ability of the RH strain, we further investigated TGGT1_286090′s differentiation function in the Type II Pru strain. Consistent with the RH strain results, PruΔ*286090′*s differentiation ability was significantly reduced (*p* < 0.001). Given TGGT1_286090′s effect on growth and virulence, we speculate that its deletion affects the translation of mRNAs involved in differentiation and growth, resulting in the observed phenotype. While this manuscript was under revision, Wang F. et al. found that disruption of the TGGT1_286090 gene failed to up-regulate bradyzoite induction factors (BFD1 and BFD2) during stress-induced differentiation [29], confirming TGGT1_286090 as a key gene for the parasite differentiation.

These studies highlight the TGGT1_286090 gene as a key regulator of translation, essential for *T. gondii*’s developmental stage transitions. Understanding these mechanisms can enhance our understanding of eIFs’ functions during the *T. gondii* stage conversion. The transformation from tachyzoites to bradyzoites is critical for *T. gondii* pathogenesis and long-term (latent) infection, and it is a vital strategy for evading host immunity. Thus, the TGGT1_286090 may represent a potential target for developing anti-*Toxoplasma* vaccines and drugs.

## 4. Materials and Methods

### 4.1. Parasite Strain and Cell Cultures

The tachyzoites of *T. gondii* strains RHΔ*ku80*, PruΔ*ku80,* and RHΔ*eIFs* were maintained in human foreskin fibroblast monolayers (HFFs, ATCC, Manassas, VA, USA) at 37 °C in a 5% CO_2_ atmosphere. HFF cells were cultured in Dulbecco’s Modified Eagle Medium (DMEM) supplemented with 2% fetal bovine serum (FBS), 10mM HEPES (pH 7.2), 100 Ug/mL streptomycin, and 100 U/mL penicillin [30]. Infected cells were passed through a 27-gauge needle to release tachyzoites and filtered using a 5 μm polycarbonate membrane filter as previously described [10].

### 4.2. Construction of C-Terminal-Tagged Strains

To examine the expression and localization of eIFs in the RHΔ*ku80* strain, we performed C-terminal tagging of six eIFs genes. The CRISPR-Cas9 plasmids targeting the 3′ untranslated region (UTR) of each gene were generated following established protocols [10,31,32]. A CRISPR plasmid specific to the region near the STOP codon of each eIF gene was prepared, and the 6 × HA and DHFR (dihydrofolate reductase) fragment was amplified from the pLIC-6 × HA-DHFR plasmids using gene-specific primers. One primer contained 42 bp of the 3′ region of the eIF gene (excluding the STOP codon), while the other primer included 42 bp of the eIF gene following the corresponding SgRNA (Supplementary Table S1). The purified fragment was co-transfected with the eIFs-specific CRISPR-Cas9 plasmids into freshly released *T. gondii* RHΔ*ku80* tachyzoites via electroporation. Positive clones were validated by PCR, followed by verification using immunofluorescence assay (IFA) and Western blotting.

### 4.3. Construction of eIF Mutant and Complemented Strains

The six eIF genes of RHΔ*ku80* strains were deleted using CRISPR-Cas9-mediated homologous recombination approach as previously described [10,33]. A single specific SgRNA targeting each eIF gene was incorporated into the pSAG1-Cas9-SgUPRT plasmid to replace the UPRT gRNA using the Q5 Mutagenesis Kit (NEB). The DHFR-resistant sequence and pUC19 plasmid portion were amplified from pUPRT-DHFR-D and pUC19 plasmids, respectively. The 5′ UTR and 3′ UTR homologous arms for each eIF gene were derived from *T. gondii* genomic DNA using specific primers. These DNA fragments were then fused into the pUC19 plasmid using the CloneExpress II one-step Cloning Kit (Vazyme) for multi-fragment cloning. Once sequencing validation was completed, the positive plasmid served as a template for amplifying the 5′-UTR -DHFR-3′-UTR homologous regions. Approximately 40 μg of the targeted pSAG1-Cas9-SgeIFs knockout plasmids and 25 μg of the 5′HR-DHFR-3′HR templates were purified and co-transfected into *T. gondii* RHΔ*ku80* or PruΔ*ku80* tachyzoites via electroporation. Subsequent selection with 3 μM pyrimethamine enabled isolation of a single strain by limiting dilution in 96-well plates. The knockout eIF strains were confirmed by diagnostic PCRs (PCR1, PCR2, and PCR3) and RT-PCR using the primers detailed in Supplementary Table S2.

To construct a complemented strain of PruΔ*286090*, the complete coding region of the TGME49_286090 gene was amplified, and peIFs::eIFs::Cat plasmids were constructed as previously described [33]. The primers used are listed in Supplementary Table S3. Approximately 40 μg of the pSAG1::Cas9-U6::sgUPRT plasmid was co-transfected with approximately 25 μg of the positive complementary fragment into freshly egressed PruΔ*286090* tachyzoites. The transfected cells were isolated by limiting dilution, cultured in medium containing 20 μg/mL chloramphenicol acetyltransferase, and verified by immunofluorescence staining.

### 4.4. Immunofluorescence Assay and Western Blotting Analyses

For the immunofluorescence assay (IFA), HFF cells were infected with RH strain tachyzoites. After 24 h, the infected cells were fixed with 4% paraformaldehyde (PFA) and permeabilized with 0.1% Triton X-100 for 20 min. The cells were then treated with primary antibodies: rabbit anti-IMC1 (1:500) and mouse anti-HA (1:500) (Invitrogen, Thermo Fisher Scientific, Waltham, MA, USA), overnight at 4 °C. Secondary antibodies, Alexa Fluor 488 anti-rabbit IgG (1:1000) and Alexa Fluor 594 goat anti-mouse IgG (1:1000), were then applied at 37 °C for 1.5 h. Imaging was performed using a Leica confocal microscope system (TCS SP8, Leica, Wetzlar, Germany).

For Western blotting, freshly purified tachyzoites were incubated on ice with RIPA lysis buffer containing protease inhibitor and EDTA for 30 min. The resulting supernatants were subjected to SDS-PAGE analysis and transferred onto a PVDF membrane. Primary antibodies, rabbit anti-Aldolase (1:500) and rabbit anti-HA (1:1000), were used followed by goat anti-rabbit HRP (1:10,000) as the secondary antibody. Protein bands were visualized using an ECL chemiluminescence kit (Thermo Fisher Scientific, Waltham, MA, USA).

### 4.5. Plaque Assay

To examine the capacity of eIF knockout strains to form plaques, approximately 200 tachyzoites of both RHΔ*eIFs* and wild-type (WT) strains were introduced into 12-well plates containing a monolayer of HFF cells, as previously described [34]. After 7 days of incubation at 37 °C in a 5% CO_2_ environment, the medium was removed and the infected cells were fixed with 4% PFA for 30 min, followed by staining with 0.5% crystal violet for 20 min at room temperature. The quantity and size of the plaques were examined and recorded.

### 4.6. Intracellular Replication and Egress Analyses

To investigate whether eIF genes play a role in the parasite intracellular replication and egress, newly released tachyzoites of RHΔ*eIF* and WT strains were added to cell culture dishes containing HFF monolayers for 1 h. Approximately 1 × 10^5^ tachyzoites from each eIF knockout or WT strain were used per dish as previously described [30,35]. After discarding the medium, the plates were washed thrice to remove extracellular parasites. Infected cells were fed with fresh culture medium and kept at 37 °C in a 5% CO_2_ atmosphere for 24 h. HFF cells were then fixed for 30 min with 4% PFA. After washing thrice with PBS, the cells were permeabilized with 0.1% Triton X-100 for 20 min and immunostained with mouse anti-SAG1, followed by Alexa Fluor 594 goat-anti mouse IgG. A minimum of 100 parasitophorous vacuoles (PVs) were randomly chosen for analysis, and the number of parasites within the PVs was documented.

For the egress assay, plates with a monolayer of HFF cells were infected by tachyzoites of RHΔ*eIF* or WT strains, at approximately 1 × 10^5^ tachyzoites per plate, and were incubated at 37 °C in a 5% CO_2_ environment for 1 h. The dishes were rinsed three times with PBS to remove free-floating parasites and then supplemented with fresh culture medium for an additional 36 h. The treated cells were exposed to 3 μM calcium ionophore A23187 in DMEM. The timing and visualization of parasite egress from host cells were captured using live cell microscopy [35].

### 4.7. Invasion Assay

To examine whether the disruption of eIF genes affects the parasite invasion, 2 × 10^6^ tachyzoites (RHΔ*eIF* and WT strains) were used to infect a 12-well cell culture plate (Thermo Fisher Scientific) at 37 °C for 30 min. The medium was then removed and 4% PFA was added. Extracellular parasites were stained with mouse anti-SAG1 antibody (1:500) at 37 °C for 2 h, and then Alexa Fluor goat anti-mouse antibody 594 (1:500) was added for 1 h at 37 °C. After washing three times with PBS, infected HFF monolayers were permeabilized with 0.1% Triton X-100 for 30 min. Cells were then treated with rabbit anti-GAP45 antibodies (1:500) at 37 °C for 2 h, and Alexa Fluor goat-rabbit antibody 488 (1:500) was used at 37 °C for 1 h, as previously described [28,29,30]. A fluorescence microscope was used to visualize tachyzoite invasion, all tachyzoites were green, and the uninvaded tachyzoites were red.

### 4.8. Virulence Assessment during Acute and Chronic Infection

Specific-pathogen-free (SPF) Kunming mice (female, 6–8 weeks old) were purchased from the Center of Laboratory Animals, Lanzhou Veterinary Research Institute, Chinese Academy of Agricultural Sciences. Every effort was made to minimize the number and suffering of the animals in the experiment. The mice were kept in a regulated environment, with a steady 12 h dark/light cycle and a humidity level between 50–60%. They were given a week to acclimate to prevent any external stress responses. The mice were then infected with tachyzoites of either RHΔ*eIF* or WT strains (6 mice per strain) by intraperitoneal (i.p.) injection, with each mouse receiving 100 tachyzoites. All mice were observed daily to monitor the progression of illness and humane endpoints [32,34].

### 4.9. Assessment of Bradyzoite Differentiation

To evaluate the ability of the eIF knockout strain to form cysts in vitro, monolayers of HFF cells were infected with WT or knockout tachyzoites of Type I RH or Type II Pru strains as previously described [30]. HFF cells were cultured at 37 °C with 5% CO_2_ for 2 h, and extracellular parasites were removed. Alkaline DMEM medium (pH 8.2) was added to dishes in a CO_2_-free incubator at 37 °C, with medium changes every 24 h to maintain the alkaline condition [10,36]. After 72 h, samples were fixed with 4% PFA for 30 min and stained with rabbit anti-IMC1, followed by FITC-labeled *Dolichos biflorus agglutinin* (DBA) and Alexa Fluor 594 goat anti-rabbit IgG stain. Bradyzoite differentiation was examined using a Leica confocal microscope system (TCS SP8, Leica, Wetzlar, Germany) and at least 200 PVs were randomly counted as previously described [36,37].

### 4.10. Statistical Analysis

Experimental data obtained both in vitro and in vivo were analyzed using GraphPad Prism 9 software (GraphPad Software, La Jolla, CA, USA). Results from three separate experiments were presented as means ± standard deviations (SD). Two-tailed, unpaired Student’s *t*-test and one-way analysis of variance (ANOVA) were utilized to determine significant differences between two groups or three or more groups, respectively. Statistical significance was defined as a *p*-value less than 0.05. The significance levels are denoted on the figures by asterisks as follows: *, *p* < 0.05; **, *p* < 0.01; ***, *p* < 0.001; ****, *p* < 0.0001.

## 5. Conclusions

We generated gene deletion strains and C-terminal epitope tags for six eIF genes in the *T. gondii* Type I RH strain. Our data showed that the TGGT1_286090 gene is partially responsible for *T. gondii* invasion and virulence and plays a critical role in cyst formation in vitro. Further studies are needed to elucidate the regulatory mechanisms controlling the influence of the TGGT1_286090 gene on bradyzoite differentiation, which may lead to new strategies for controlling the most persistent stage of *T. gondii* and combatting latent toxoplasmosis.

## Data Availability

The original contributions presented in this study are included in the article. Further inquiries can be directed to the corresponding authors.

## Supplementary Materials

The following supporting information can be downloaded at https://www.mdpi.com/article/10.3390/ijms25147834/s1.
