# Supplementary material for: Functional Characterization of Six Eukaryotic Translation Initiation Factors of Toxoplasma gondii Using the CRISPR-Cas9 System
Source: Int J Mol Sci. 2024 Jul 17;25(14):7834. doi: 10.3390/ijms25147834 (PMC11276994; doi:10.3390/ijms25147834)
Supplement: Supplementary file 1 [file ijms-25-07834-s001.zip › Figure S1.pdf]

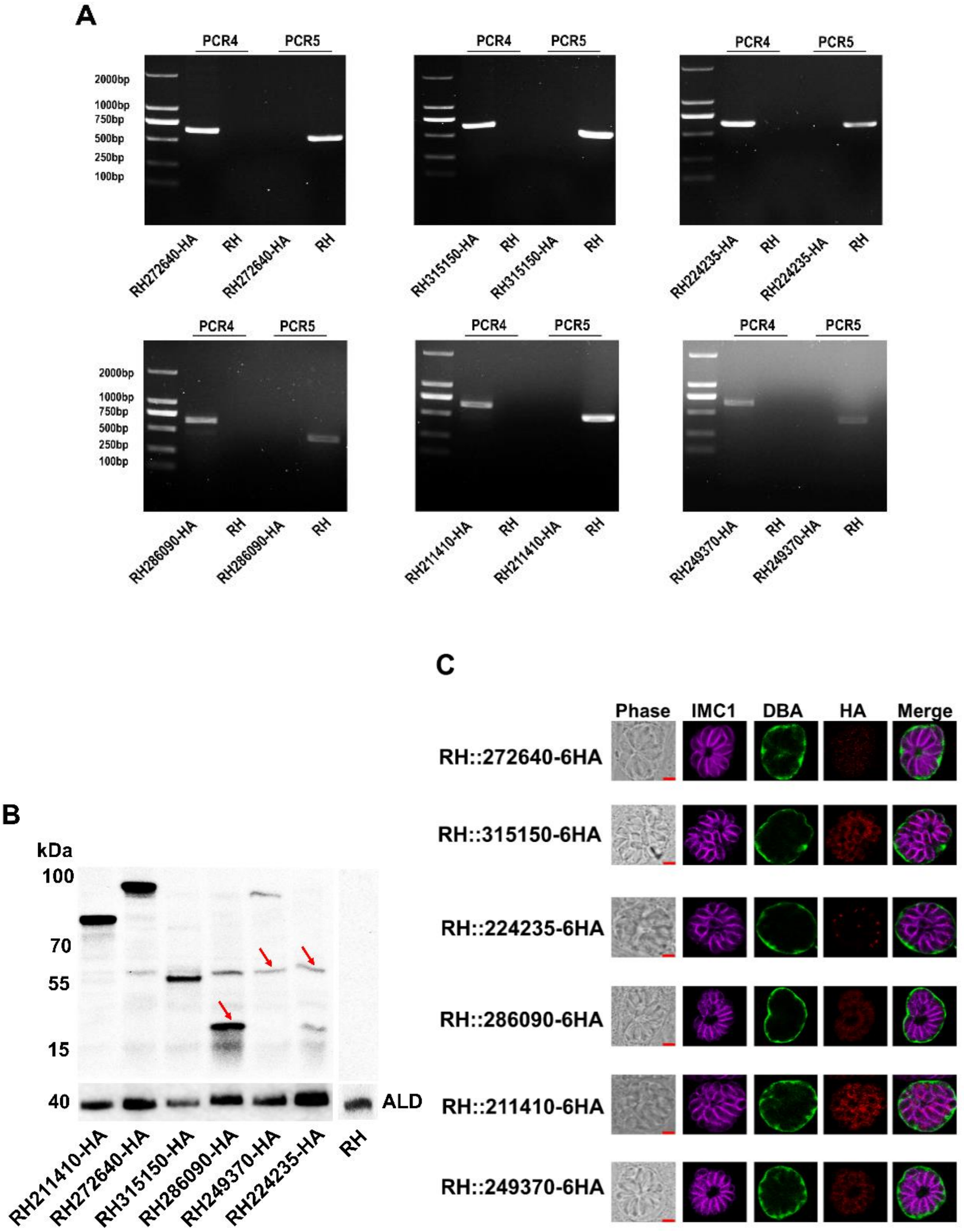

Figure S1 Construction and validation of six *T. gondii* RH::eIFs-HA strains. (A) Verification of six RH*eIFs*-HA strains by diagnostic PCRs. PCR4 was designed to detect the insert of six hemagglutinin (6×HA). PCR5 was designed to detect the successful replacement of C-terminal *eIFs* genes by 6×HA fragment. (B) Western blotting was performed to confirm the expression of the six RH*eIFs*-HA strains and the wild RH strain was used as a negative control. Anti-aldolase (ALD) served as a loading control. Red arrows indicate the target protein. (C)The infected cells were then cultured under stress condition in alkaline medium for 48 h at 37°C in ambient air. After 2 days, bradyzoites differentiated from tachyzoites were stained with anti-HA epitope (red), anti-IMC1(purple) and DBA-FITC (green). Scale bars, 3 µm.
