# Supplementary material for: Functional Characterization of Six Eukaryotic Translation Initiation Factors of Toxoplasma gondii Using the CRISPR-Cas9 System
Source: Int J Mol Sci. 2024 Jul 17;25(14):7834. doi: 10.3390/ijms25147834 (PMC11276994; doi:10.3390/ijms25147834)
Supplement: Supplementary file 1 [file ijms-25-07834-s001.zip › Figure S2.pdf]

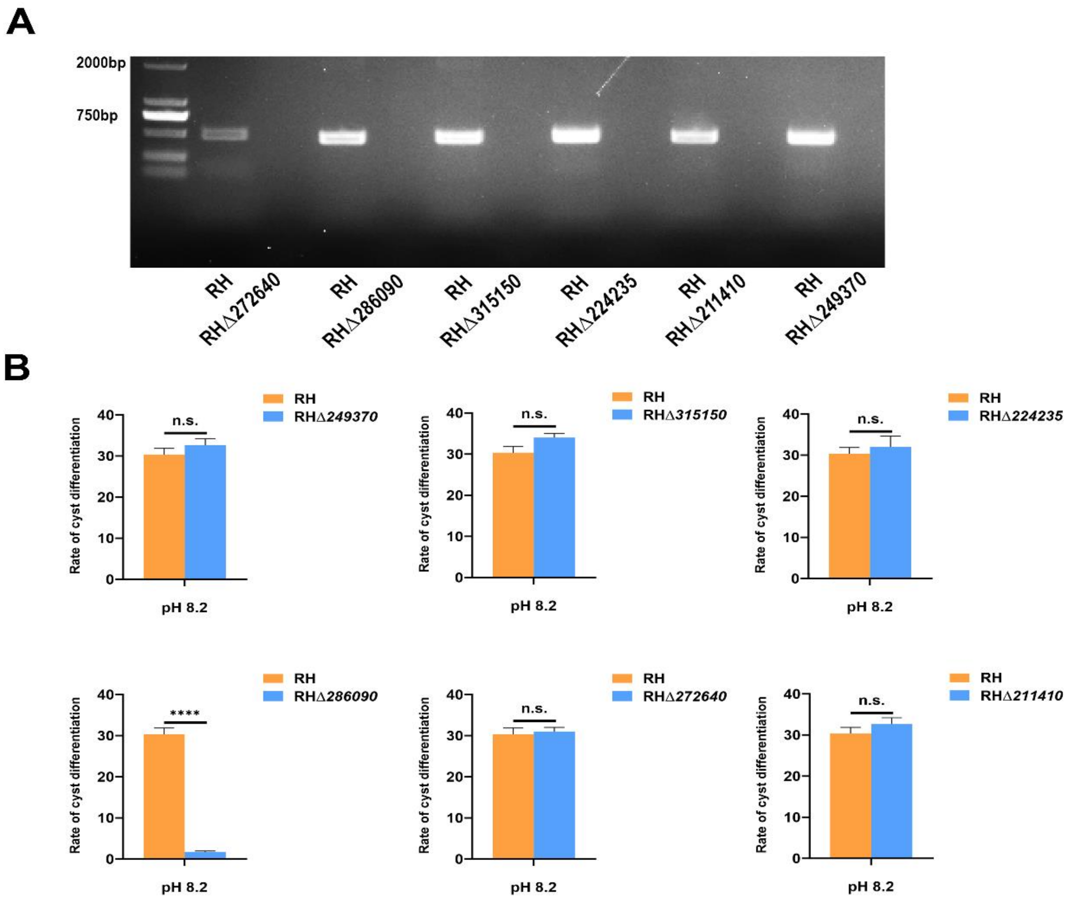

Figure S2 The RHΔ*eIFs* strain was verified by RT-PCR and effect of eIFs deletion on cyst formation *in vitro*. (A) RHΔ*eIFs* were successfully constructed at cDNA level. (B) Cyst conversion rates of RH and RHΔ*eIFs* strains. At least 100 vacuoles were counted in each experiment. Experiments were divided into cyst wall positive vacuoles (DBA-positive) and normal vacuoles (DBA-negative). By Student's t test, there was no significant difference in the conversion rates of the five RHΔ*eIFs* strains ($p > 0.05$), compared with the WT strain. Only the rate of cyst formation in RHΔ*286090* strain was significantly decreased in vitro ($p < 0.0001$).
