# Supplementary material for: Functional Characterization of Six Eukaryotic Translation Initiation Factors of Toxoplasma gondii Using the CRISPR-Cas9 System
Source: Int J Mol Sci. 2024 Jul 17;25(14):7834. doi: 10.3390/ijms25147834 (PMC11276994; doi:10.3390/ijms25147834)
Supplement: Supplementary file 1 [file ijms-25-07834-s001.zip › Table S1.pdf]

Table S1: Primers used in the construction of the epitope-tagging strains.

	sgRNA	HR-primers (5′-3′)	KO-primers (5′-3′)
TGGT1_315150	ATCCGAGAGAGGCCTGAGAG	Forward:GAAGCAGAGGAGGCACCGGAGAAGAAGAATCCGAGAGAGGCC	Forward:CGCCTCTCTCGGTTCTTCTG
		Reverse:ACGGCTAGCCGACAGTTCCGCCGCGTCGCGCGACGTCCACTC	Reverse:TCCAGACACCCATGAGGAAA
TGGT1_272640	AGAACGTCAAGTAAGAAGC	Forward:AACCCGCGGCTGGTCGCGTTCTGCGAGTGGCTGGCCCACGAA	Forward:ATTGTAAATCCTCTCTAC
		Reverse:GTTCCTTTCCTAACACACGGCAGAGGCAGATATCCACCAGCT	Reverse:GCAAATAGTCTCAATCTG
TGGT1_224235	GTCTCGTCGGAAGGCTGACA	Forward:ACAGAGGACCTCCGTGAGTCTGTCGAAGTCTCGTCGGAAGGC	Forward:CGTTAAAGAAATCTTGGT
		Reverse:ATAAAAGCCTGAGAAGCTTCACTGATAAGGAGATCACCGTGTT	Reverse:ACAATTAACAGCAGTAAG
TGGT1_286090	ATTCATGGTGCCTAAATAGC	Forward:GAAGCAATCTGCAGTGTCGACCAGATCCGCATTCATGGTGCC	Forward:TATATTCGTGTATCTTGAGT
		Reverse:TCTTTTCGCGCATCAGAAACCGCCTTACGTGCACTTCCTGCT	Reverse:TTTCTGTTCACATGACAT
TGGT1_249370	GCGAAAGTGAACTCCTCTCG	Forward:GTTGTCGTTGAACAAAAGAAATCGAAGGCTGGATCGCGAAAG	Forward:ATGTATCTCATTTGAATG
		Reverse:ATGCTTTCCGCCCAGGCGTTTAACAAGGAACAGCCACCACGA	Reverse:GTCTACCCATCTATATTC
TGGT1_211410	CATCGCCGCATGACGTTCAC	Forward:GAAGCCATCTGTTTGGGGGAACAAATCCGAATCCATGGGGCG	Forward:GGAGGAAAGAGATCAACTG
		Reverse:AGATCGCACTCGATTCTGTTTTGAAGAAATGCGACGCCGGTG	Reverse:GGAGAAGAAAGAAAGAAGA
