# Supplementary material for: Functional Characterization of Six Eukaryotic Translation Initiation Factors of Toxoplasma gondii Using the CRISPR-Cas9 System
Source: Int J Mol Sci. 2024 Jul 17;25(14):7834. doi: 10.3390/ijms25147834 (PMC11276994; doi:10.3390/ijms25147834)
Supplement: Supplementary file 1 [file ijms-25-07834-s001.zip › Table S2.pdf]

Table S2: Primers used in the construction of the *eIFs* genes knockout strains

Gene	sgRNA	KO-primers (5′-3′)	U5-Gibson-primers (5′-3′)	U3-Gibson-primers (5′-3′)	CDS-Gibson-primers (5′-3′)
TGGT1_315150	GCGGCGGAAGCTGGAGTCTC	Forward: ATCAAGATTTCTTCTATT<br>Reverse: TCTCTCCATAGATATCAG	Forward: GGAACTTGTCTCTCTGTTTG<br>Reverse: CTCCTTCTCTGTCTCCTC	Forward: GAAGGCAAACAGGAAACA<br>Reverse: CCTCTAAATCCCTCGTCC	Forward: GAAAGGCTGCTGGCCGCTA<br>Reverse: AACTCTCTCCGTTTTCCT
TGGT1_272640	GTGAGCGACGATCGAGAGGA	Forward: TATATGTAATATATGCTTT<br>Reverse: ATATGGTAAGTAATCATA	Forward: AACGCTTGAATTCTGAGA<br>Reverse: ACTCCATGTGTAGTTTCG	Forward: TGGTCAGAATGCCTTATG<br>Reverse: CCAGCTCAACAAGAAACC	Forward: GTGGGAGACATCCACGAGG<br>Reverse: ACCTCAGGGACGATGGGGT
TGGT1_224235	GATTCGAAGTGGCAACAACG	Forward: CTCATTGTTGTTCATAAC<br>Reverse: GATTCAAGCTCAGTCTAG	Forward: TATCTATGTGCATCTAAT<br>Reverse: TTTTCAGTAAAGAGTAAA	Forward: GTTAAAGAAATCTTGGTG<br>Reverse: TATATGCGATAACGAATA	Forward: TTGAAGAAAGACGCTGGG<br>Reverse: AAAGGTGTCAGGTCGCTTCTC
TGGT1_286090	GAAGAGCGTGACGACGGTTC	Forward: TCGTAAGTTGAGAAGTAA<br>Reverse： TATTTAGGCACCATGAAT	Forward: CTTTACTTCTCAACTTAC<br>Reverse: ACAATTCATTTACAAGAT	Forward: CTTTACTTCTCAACTTAC<br>Reverse: CTCACTGAAACAAATATC	Forward: ATGTCACTCGACATTCAAAAC<br>Reverse: TTAGGCACCATGAATGCGGA
TGGT1_249370	GCATAGAAGTCATGCAGCTC	Forward: GGAAGTGTCAGGTGTATG<br>Reverse: CTCTCTTTCTCACCCTCTC	Forward: CAGGATTCGCAAAACAAG<br>Reverse: CACTCATTCACCAAACAG	Forward: CGTTGAACAAAAGAAATCG<br>Reverse: AGAGTGAAGAGCAAATG	Forward: GCACACCCAAGGTCGAGA<br>Reverse: TGTTTTTGGGCGTCGAAAA
TGGT1_211410	GTGTATGTCGTTCTGATTCG	Forward: TCTCCATTAGGTTAATTG<br>Reverse: TAGTATTGGAAAAGCATA	Forward: AGGGTTTGTGTTCATCA<br>Reverse: GAGACCGAAAAGACAGA	Forward: CATAATTTCGGTATTTCC<br>Reverse: AAAGAGAAGATAAACAAC	Forward: GAAGTGCCCGGACTTGCTGCC<br>Reverse: CGCCCTCTTCCTCGCCATTT
