# Supplementary material for: Functional Characterization of Six Eukaryotic Translation Initiation Factors of Toxoplasma gondii Using the CRISPR-Cas9 System
Source: Int J Mol Sci. 2024 Jul 17;25(14):7834. doi: 10.3390/ijms25147834 (PMC11276994; doi:10.3390/ijms25147834)
Supplement: Supplementary file 1 [file ijms-25-07834-s001.zip › Table S3.pdf]

Table S3: Primers used in the construction of the *eIFs* genes' complemented strains

Primer	Sequence (5′-3′)	Application
peIFs-F	AGTGGAGGACGGGAATTCGGGCCCGTTAGGGGAGCGTTCTCCGAACA	To construct the start codon of eIFs
peIFs-R	GAAGTTTTGAATGTCGAGTGACAT	
eIFs-CDS-F	ATGTCACTCGACATTCAAAACTTC	To construct the CDS of eIFs
eIFs-CDS-R	GGTCGAGCCCGAGCCCTTGCTAGCGGCACCATGAATGCGGATCTGGTC	
3HA-Cat-Gbison-F	TACACGAGAGACCGGATTGGTTGTATGCCTCTCTTAGACCAACGAAGC	Amplification of 3xHA-Cat fragment to construct PeIFs::eIFs::Cat plasmid
3HA-Cat-Gbison-R	GGTCGAGCCCGAGCCCTTGCTAGCAGAAAGAGAAATCTCAAGTTCTCG	
UPRT-Pro-KZ-F	GCTGCAGCGGCCGCACAGTTTTTCGACTTGCTCAAGTTG	Amplification of 3xHA-Cat fragment to construct peIFs::eIFs::Cat plasmid
UPRT-Ter-KZ-R	GCACACGATGCACGGCGCCGGCAC	
SgRNA-eIFs-epitope tag	GCTGCAGCGGCCGCAGCTGCCTACAACAGCGTGGAGACATTTGAG	Amplification of peIFs::eIFs::Cat fragment to construct PruΔ*eIFs-C* strain
